# A blend of functional amino acids and grape polyphenols improves the pig capacity to cope with an inflammatory challenge caused by poor hygiene of housing conditions

**DOI:** 10.1186/s12917-023-03580-w

**Published:** 2023-01-30

**Authors:** Alícia Zem Fraga, Paulo Henrique Reis Furtado Campos, Luciano Hauschild, Tristan Chalvon-Demersay, Martin Beaumont, Nathalie Le Floc’h

**Affiliations:** 1 ‬PEGASE, INRAE, Institut Agro, 35590 Saint Gilles, France; 2 Department of Animal Science, São Paulo State University (Unesp), School of Agricultural and Veterinarian Sciences, Jaboticabal, São Paulo, 14884-900 Brazil; 3 Department of Animal Science, Universidade Federal de Viçosa, Viçosa, Minas Gerais 36570-900 Brazil; 4 METEX ANIMAL NUTRITION, 32 Rue Guersant, 75017 Paris, France; 5 GenPhySE, Université de Toulouse, INRAE, ENVT, 31326 Castanet-Tolosan, France

**Keywords:** Health, Inflammation, Weaning Transition

## Abstract

**Background:**

Dietary supplementation with a blend of functional amino acids (AA) and grape extract polyphenols contributes to preserve intestinal health and growth performance of piglets during the post-weaning period. In the present experiment, we assessed if a supplementation with a mix of AA and grape extract polyphenols during the post-weaning period would persist to improve the pig capacity to cope with a subsequent challenge caused by poor hygiene of housing conditions. Eighty pigs weaned at 28 days of age were fed a standard diet supplemented (AAP) or not (CNT) with 0.2% of a blend of AA (glutamine, arginine, cystine, valine, isoleucine, and leucine) and grape extract polyphenols during the post-weaning period (from week 0 to 6). At week 6, pigs were transferred to a growing unit where 50% of pigs previously fed AAP and CNT diets were housed in good and the other 50% in poor hygiene conditions for 3 weeks (from week 7 to 9; challenge period). All pigs were fed a standard growing diet that was not supplemented with AAP. We measured pig growth performance, plasma indicators of inflammation, digestive integrity, and oxidative status, and scored fecal consistency. Differences were considered significant at *P* ≤ 0.05.

**Results:**

One week post-weaning, pigs fed AAP had lower plasma concentrations of haptoglobin than CNT pigs (*P* = 0.03). Six weeks post-weaning, plasma concentrations of diamine oxidase (DAO) were lower (*P* = 0.03) whereas those of vitamin E and A were greater (*P* ≤ 0.05) in pigs fed AAP compared to CNT pigs. The prevalence of diarrhea was higher in CNT pigs compared to AAP pigs (*P* < 0.01). During the challenge period, only pigs previously fed CNT diet had lower growth rate in poor than good conditions (*P* ≤ 0.05). They had also greater plasma concentrations of haptoglobin and oxidative stress index (OSI) and lower plasma concentrations of vitamin E in poor than good hygiene conditions (*P* ≤ 0.05).

**Conclusions:**

Pigs fed AAP diet during post-weaning had less diarrhea and plasma concentrations of a digestive integrity marker, as well as greater plasma concentrations of antioxidant indicators during the post-weaning period. The beneficial effects of AAP supplementation persisted after the post-weaning period as evidenced by the absence of effects of the hygiene challenge on growth and health indicators in pigs previously fed APP. This clearly indicated a greater ability of pigs fed AAP to cope with the poor hygiene conditions.

**Supplementary Information:**

The online version contains supplementary material available at 10.1186/s12917-023-03580-w.

## Background

In conventional pig farming systems, pigs are usually weaned between 3 and 5 weeks of age, when they still have an immature immune system and low digestive capacity [1]. Furthermore, during the weaning transition pigs are exposed to multiple stressors including changes of the diet, separation from their mothers and mixing with unfamiliar littermates [2, 3]. The changes predisposed the animals to reduced feed intake, impaired intestinal functions, and low nutrient absorption, all contributing to post-weaning diarrhea and poor growth [3]. The post-weaning period is thus considered as one of the most critical and stressful period for the pig. Nutritional strategies have been widely studied to support immune maturation and enhance gastrointestinal morphology and functions of pigs at weaning.

Besides being building blocks of proteins, amino acids (AA) are known to be nutrients that contribute to health maintenance. Functional AA was defined as those AA that exert functions other than being precursors of proteins [4]. Dietary supplementation with arginine (Arg), branched-chain amino acids (BCAA; leucine, isoleucine, and valine), cystine (Cys2), and/or glutamine (Gln) separately [5–8] or in a blend [9] was associated with improvement of intestinal integrity and immune status in weaned and/or inflammatory-challenged pigs. Furthermore, plant extracts containing proanthocyanidins, the most common and consumed dietary polyphenols, have been increasingly studied as potential health-promoting dietary components. Previous findings have demonstrated a reduced prevalence of post-weaning diarrhea in piglets fed with a grape extract polyphenol supplemented diet by the improvement of the immune and antioxidant capacities [10]. Recently, a study reported that diet supplementation with a blend of functional AA and grape extract polyphenols improved growth performance of piglets during the post-weaning period [11]. This beneficial effect was associated with a greater production of protective bacterial metabolites, a higher abundance of *Lactobacillus* in the jejunum and a lower abundance of *Proteobacteria* in the caecum [11]. Therefore, this study was performed to evaluate whether 0.2% of a blend of functional AA (Arg, Gln, Cys2 and BCAA) and grape extract polyphenols (AAP) supplied during the post-weaning period may improve the pig capacity to cope with the weaning transition and with an inflammatory challenge caused by poor hygiene conditions in the beginning of the growing period.

## Results

Eighty pigs were weaned at 28 days of age and fed a control standard diet supplemented or not with 0.2% of a blend of functional amino acids and grape polyphenols (AAP and CNT diets, respectively) during the post-weaning period (from week 0 to 6). Six weeks after weaning (week 6), pigs were transferred in a growing unit where they were housed in good or poor hygiene conditions and fed a standard diet for 3 weeks (challenge period). The finishing period lasted 11 weeks and all animals were slaughtered at the end of the experiment (week 20; at 24 weeks of age). Two experimental groups, AAP and CNT pigs, were compared during the post-weaning period and four experimental groups were compared during the challenge period: AAP-good, AAP-poor, CNT-good and CNT-poor for pigs previously fed AAP housed in good and poor hygiene conditions or CNT housed in good and poor hygiene conditions.

### General observations

Data from 80 pigs were kept for statistical analyses. One CNT-poor pig died probably due to intestinal torsion during the finishing period. Average ambient temperatures were 23.5 ± 1.5 °C (post-weaning period), 20.9 ± 1.0 °C and 21.1 ± 1.5 °C (in good and poor conditions, respectively), and 19.6 ± 1.0 °C (finishing period). Lower air concentration of carbon dioxide (625 vs 850 ppm; *P* < 0.01) and ammonia (7.5 vs 10.6 ppm; *P* = 0.08) levels were observed in good as compared to hygiene conditions. The concentration of hydrogen sulfide in the air did not differ between hygiene conditions (0.50 and 0.53 ppm in good and poor conditions, respectively; *P* = 0.92).

**Fig. 1:**
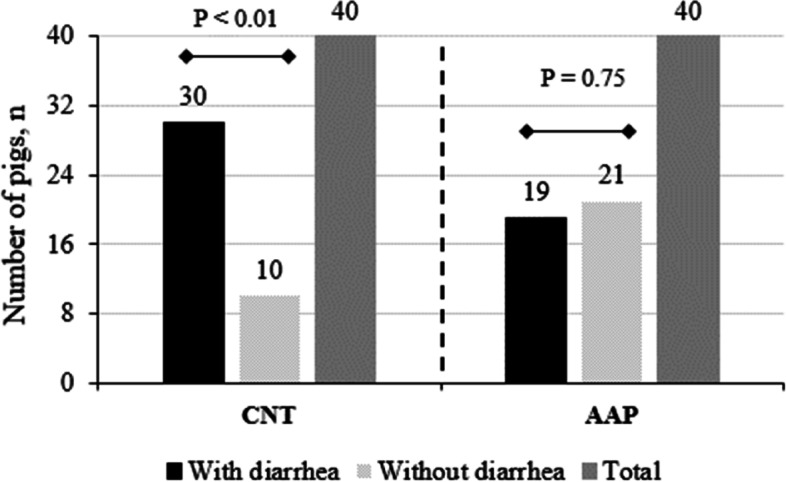
Prevalence of diarrhea in CNT and AAP pigs during the post-weaning period^1,2^. With diarrhea: Pigs with diarrhea at least once; Without diarrhea: Pigs with no diarrhea (sum of animals with score levels 0 and 1); Total: Total of pigs per diet (CNT and AAP, *n* = 40). P: Probability value for the effect of groups (with and without diarrhea) for each diet. Values were compared using a χ^2^ test. ^1^ Post-weaning period: Pigs received a CNT or AAP diet during the pre-starter (from week 0 to 1) and starter (from week 2 to 6) phases. The AAP diet consisted of a control standard diet (CNT) supplemented with 0.2% of a blend of functional amino acids (L-arginine, L-cystine, L-leucine, L-valine, L-isoleucine, and L-glutamine) and grape polyphenols. ^2^ Data collected three times per week (from week 0 to 6)

During the post-weaning period, the following clinical signs were recorded: thinness [AHOL_0003136; [12]] and apathy [AHOL_0003037] (three CNT pigs), sneezing [AHOL_0003116] and cough [AHOL_0003090] (four AAP and five CNT pigs), nose wound (one AAP pig), and diarrhea [AHOL_0003105] (21 AAP and 30 CNT pigs). In CNT pigs, the number of pigs with diarrhea was greater than the number of pigs without diarrhea (*P* < 0.01; Fig. 1) whereas it did not differ in AAP pigs (*P* = 0.75). During the challenge period, the following clinical signs were observed: umbilical hernia (one CNT-good pig), sneezing and cough (12 pigs: three AAP-good, four CNT-good, two AAP-poor and three CNT-poor), and diarrhea (43 pigs: one AAP-good and two CNT-good; whereas all pigs had diarrhea at least once in poor conditions). The prevalence of diarrhea did not differ between pigs previously fed AAP and CNT (*P* = 0.53), whereas it was lower in good as compared to poor hygiene conditions (*P* < 0.01). Clinical signs as thinness and apathy (one pig CNT-good), diarrhea (one pig CNT-poor), umbilical hernia [AHOL_0003267] (one pig AAP-good and one AAP-poor), and skin wound (43 pigs: 10 AAP-good, 12 CNT-poor, 11 CNT-good and 10 CNT-poor) were observed during the finishing period.

### Growth performance

During the post-weaning period, there was no effect of the experimental diets (ED) on performance traits (Table 1). During the challenge period (from week 7 to 9), regardless of experimental diets fed during the post-weaning period, pigs housed in good hygiene conditions had lower feed conversion ratio (FCR, ATOL_0001580; [12]) compared to pigs housed in poor conditions (2.21 and 2.52 in good and poor conditions, respectively; *P* < 0.01). Interaction between ED and hygiene conditions (Hyg) was observed for final body weight (BW, ATOL_0000351) measured at week 9 (W9) and average daily gain (ADG, ATOL_000989; *P* ≤ 0.05), and a trend for interaction was observed for average daily feed intake (ADFI, ATOL_0005508; ED × Hyg: *P* = 0.07, Table 2). Whereas CNT pigs had lower final BW and ADG at W9 in poor as compared to good conditions (*P* ≤ 0.05), there was no difference between hygiene conditions for AAP pigs (*P* > 0.10). A trend for lower ADFI was observed in good compared to poor conditions in AAP pigs only (1.839 and 1.945 kg/day in good and poor conditions, respectively; *P* = 0.07).

**Table 1:** Growth performance of CNT and AAP pigs during the post-weaning period.^1^

	Experimental diets^2^		SEM^3^	*P*-value^4^
Items	CNT	AAP		
No.^5^	40	40		
Post-weaning, from week 0 to 6				
Initial BW, kg	8.7	8.9	0.12	0.60
Final BW, kg	28.3	28.1	0.33	0.75
ADFI, kg/day	0.806	0.799	0.01	0.48
ADG, kg/day	0.489	0.483	0.01	0.69
FCR	1.68	1.66	0.03	0.89

*BW* body weight, *ADFI* average daily feed intake, *ADG* average daily gain, *FCR* feed conversion ratio

^1^Values are least square means

^2^Post-weaning period: Pigs were fed the CNT or AAP diet for 3 weeks after weaning (from week 0 to 6) phases. The AAP diet consisted of a control standard diet (CNT) supplemented with 0.2% of a blend of functional amino acids (L-arginine, L-cystine, L-leucine, L-valine, L-isoleucine, and L-glutamine) and grape polyphenols

^3^Standard error of the mean

^4^Probability value for the effect of experimental diets

^5^Number of pigs per group

**Table 2:** Growth performance of CNT and AAP pigs housed in good (good) or poor (poor) hygiene of housing conditions for 3 weeks.^1^

Experimental diets^2^	CNT		AAP		SEM^4^	*P*-value^5^		
Hygiene conditions^3^	Good	Poor	Good	Poor		ED	Hyg	ED × Hyg
No.^6^	20	20	20	20				
Challenge period, from week 7 to 9								
Initial BW, kg	28.5	28.2	27.8	28.4	0.33	0.75	0.80	0.41
Final BW, kg	46.2 a	42.5 b	45.4 ab	44. 9 ab	0.56	0.55	0.03	0.04
ADFI, kg/day	1.846	1.776	1.839	1.945	0.02	0.10	0.67	0.07
ADG, kg/day	0.843 a	0.683 b	0.838 a	0.786 ab	0.02	0.14	< 0.01	< 0.01
FCR	2.21	2.58	2.21	2.45	0.03	0.60	< 0.01	0.18

*BW* body weight, *ADFI* average daily feed intake, *ADG* average daily gain, *FCR* feed conversion ratio

^1^Values are least square means

^2^Pigs were fed the CNT or AAP diet for 6 weeks after weaning (from Week 0 to 6). The AAP diet consisted of a control standard diet (CNT) supplemented with 0.2% of a blend of functional amino acids (L-arginine, L-cystine, L-leucine, L-valine, L-isoleucine, and L-glutamine) and grape polyphenols

^3^ Pigs were housed in good (good) and poor (poor) hygiene of housing conditions for 3 weeks (from week 7 to 9) and were fed a standard diet

^4^Standard error of the mean

^5^Probability values for the effect of experimental diets (ED), hygiene conditions (Hyg) and their interaction (ED × Hyg)

^6^Number of pigs per group

^a,^^b^Within a row values with different superscripts differed (P ≤ 0.05)

At the end of the finishing period, a trend for greater final BW was observed for pigs previously housed in good compared to poor conditions (128.6 and 124.5 kg in good and poor conditions, respectively; *P* = 0.08). Except for the finishing period where male had greater final BW as compared to females (130.6 and 122.2 kg for male and female, respectively; *P* < 0.01), no significant differences were observed.

### Blood indicators of inflammatory and oxidative status and gut integrity

Blood variables measured during the post-weaning period are presented in Table 3. At week 0 (W0), just before pigs were assigned to received AAP or CNT diet, there was no difference between AAP and CNT pigs for any studied variable (*P* > 0.10). One week after weaning (W1), plasma haptoglobin concentrations [ATOL_0000935] were lower (*P* = 0.03) in AAP than CNT pigs. Trends for lower values of diacron-reactive oxygen metabolites (dROM, 994.95 and 1073.23 CARRU; *P* = 0.07) and oxidative stress index (OSI, 0.38 and 0.40; *P* = 0.06) were observed in AAP than CNT pigs. At the end of the post-weaning period (W6), plasma concentrations of vitamin E and vitamin A were greater in AAP compared to CNT pigs (*P* ≤ 0.05). Lower concentrations of diamine oxidase (DAO; *P* = 0.03) and a trend for lower OSI value (0.40 and 0.43; *P* = 0.09) in AAP than CNT pigs were also observed at W6.

**Table 3:** Plasma concentrations of indicators of inflammation and oxidative status in CNT and AAP pigs measured before weaning (Week 0), and 1 and 6 weeks after weaning (Weeks 1 and 6).^1^

	Experimental diets^2^		SEM^3^	*P*-value^4^
Items	CNT	AAP		
No.^5^	40	40		
Week 0				
Haptoglobin, mg/ml	2.91	2.99	0.12	0.70
Vitamin E, µmol/L	7.78	8.23	0.37	0.54
Vitamin A, µmol/L	0.34	0.34	0.01	0.99
Diamine Oxidase, pg/ml	283.7	290.8	4.80	0.11
dROM, CARRU^6^	1000.6	994.6	17.76	0.89
BAP, µmol/L	2442.7	2472.4	23.43	0.54
ISO^7^, CARRU/µmol/L	0.41	0.40	0.01	0.23
Week 1				
Haptoglobin, mg/ml	3.52	2.98	0.13	0.03
Vitamin E, µmol/L	3.36	3.61	0.10	0.21
Vitamin A, µmol/L	0.34	0.36	0.01	0.56
Diamine Oxidase, pg/ml	312.8	316.7	6.0	0.54
dROM, CARRU^6^	1073.2	994.9	20.92	0.07
BAP, µmol/L	2327.2	2321.33	27.26	0.96
ISO^7^, CARRU/µmol/L	0.40	0.38	0.01	0.06
Week 6				
Haptoglobin, mg/ml	1.49	1.39	0.12	0.65
Vitamin E, µmol/L	1.57	2.00	0.07	< 0.01
Vitamin A, µmol/L	0.30	0.35	0.02	0.04
Diamine Oxidase, pg/ml	307.4	287.6	5.9	0.03
dROM, CARRU^6^	970.4	970.9	17.23	0.98
BAP, µmol/L	2826.4	2797.1	20.57	0.48
ISO^7^, CARRU/µmol/L	0.43	0.40	0.01	0.09

^1^Values are least square means

^2^Post-weaning period: Pigs were fed the CNT or AAP diet for 6 weeks. The AAP diet consisted of a control standard diet (CNT) supplemented with 0.2% of a blend of functional amino acids (L-arginine, L-cystine, L-leucine, L-valine, L-isoleucine, and L-glutamine) and grape polyphenols

^3^Standard error of the mean

^4^Probability value for the effect of experimental diets

^5^Number of pigs per group

^6^CARRU (Carratelli Unit, 1 CARRU = 0.08 mg H_2_O_2_/100 mL of sample)

^7^ISO (Oxidative Stress Index) = dROM/BAP

**Fig. 2:**
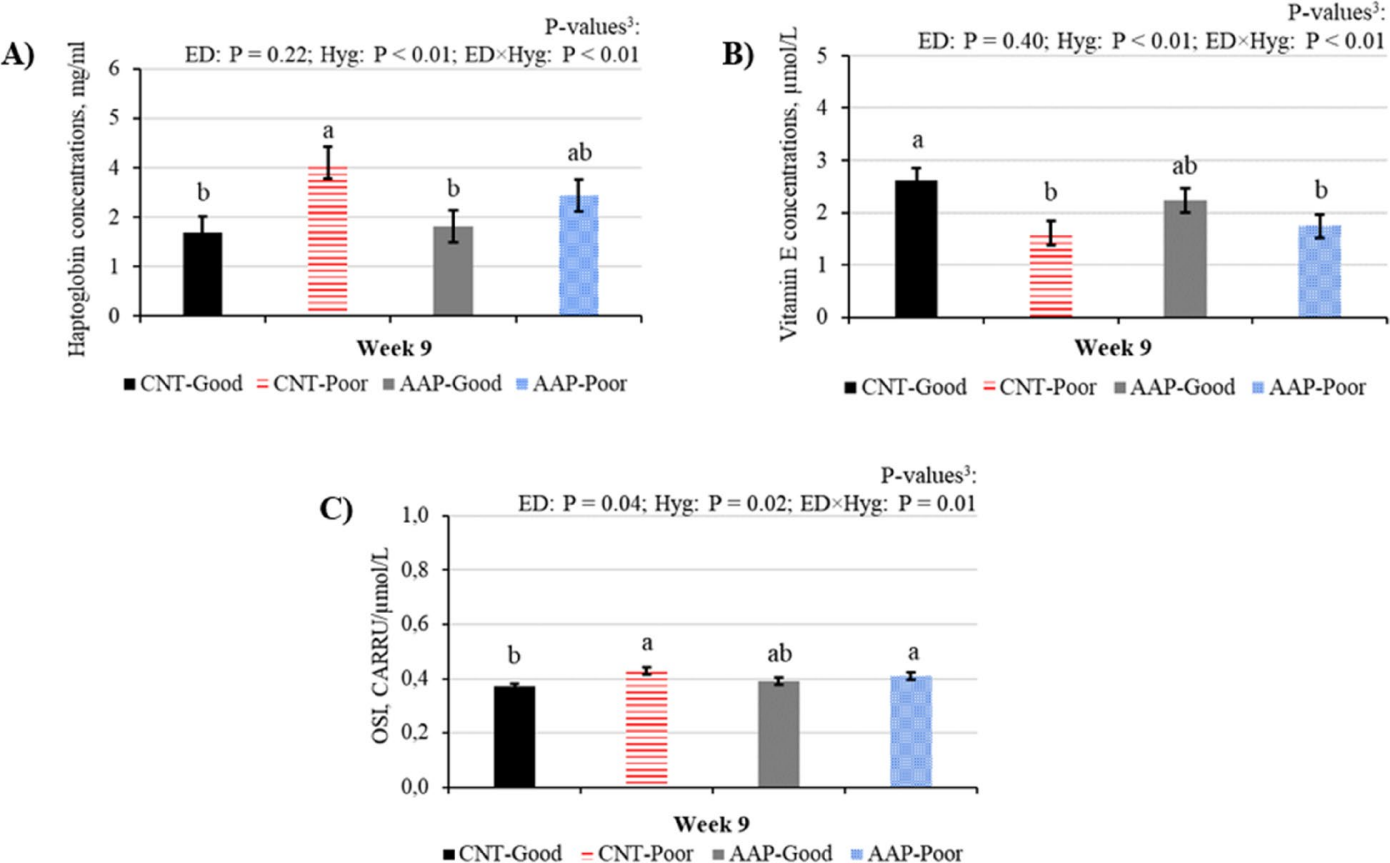
Vitamin E (A), haptoglobin plasma concentrations (B), and Oxidative Stress Index (C) at week 9.^1,2^. ^1^ Pigs received a CNT or AAP diet during the pre-starter (from week 0 to 1) and starter (from week 2 to 6) phases. The AAP diet consisted of a control standard diet (CNT) supplemented with 0.2% of a blend of functional amino acids (L-arginine, L-cystine, L-leucine, L-valine, L-isoleucine, and L-glutamine) and grape polyphenols. ^2^ After the week 6, CNT and AAP pigs were housed in good (good) and poor (poor) hygiene of housing conditions for 3 weeks (from week 7 to 9). ^3^ *P*-values: Probability values for the effect of experimental diets (ED), hygiene conditions (Hyg) and their interaction (ED × Hyg). ^a,b^ Values with different superscripts differed (P ≤ 0.05)

At the end of the challenge period (W9), pigs housed in good hygiene conditions had lower concentrations of white blood cells (WBC), granulocytes, and dROM compared to pigs housed in poor conditions (*P* ≤ 0.05; Table 4). There was an interaction between ED and Hyg for haptoglobin, vitamin E concentrations and OSI (*P* ≤ 0.05): however, differences between hygiene conditions were significant only for CNT pigs. When housed in good hygiene conditions, CNT pigs had greater concentrations of vitamin E (*P* < 0.01; Fig. 2A) and lower haptoglobin and OSI (*P* ≤ 0.05; Fig. 2B and C) than in poor conditions.

**Table 4:** Blood and plasma concentrations of indicators of inflammation and oxidative status in CNT and AAP pigs housed in good (good) or poor (poor) hygiene of housing conditions for 3 weeks^1^

Experimental diets^2^	CNT		AAP		SEM^4^	*P*-value^5^		
Hygiene conditions^3^	Good	Poor	Good	Poor		ED	Hyg	ED × Hyg
No.^6^	20	20	20	20				
Week 9								
WBC, m/mm^3^	18.51	21.49	19.56	21.85	0.48	0.34	< 0.01	0.81
Granulocyte, m/mm^3^	5.77	8.27	6.77	9.05	0.25	0.14	< 0.01	0.47
Vitamin A, µmol/L	0.49	0.48	0.49	0.41	0.02	0.43	0.16	0.24
Diamine Oxidase, pg/ml	310.8	333.4	321.9	315.5	9.06	0.81	0.57	0.31
dROM, CARRU^7^	1035	1150	1036	1148	18.6	0.97	< 0.01	0.98
BAP, µmol/L	2823	2836	2707	2800	26.1	0.15	0.26	0.37

*WBC* White Blood Cells, *dROM* Diacron Reactive Oxygen Metabolites, *BAP* Biological Antioxidant Potential

^1^Values are least square means

^2^Pigs were fed the CNT or AAP diet for 6 weeks after weaning (Week 0 to 6). The AAP diet consisted of a control standard diet (CNT) supplemented with 0.2% of a blend of functional amino acids (L-arginine, L-cystine, L-leucine, L-valine, L-isoleucine, and L-glutamine) and grape polyphenols

^3^Pigs were housed in good (good) and poor (poor) hygiene of housing conditions for 3 weeks (from week 7 to 9) and were fed the same standard diet

^4^Standard error of the mean

^5^Probability values for the effect of experimental diets (ED), hygiene conditions (Hyg) and their interaction (ED × Hyg)

^6^Number of pigs per group

^7^CARRU (Carratelli Unit, 1 CARRU = 0.08 mg H_2_O_2_/100 mL of sample)

### Fecal microbiota

The fecal microbiota was analyzed at the end of the post-weaning period (W6). There was no effect of ED on the microbiota richness and α-diversity indexes (Additional file 1). Analysis of β-diversity using the Bray–Curtis distance indicated a minor effect of ED on the microbiota structure (PERMANOVA *P* < 0.05 and 2% of explained variance R^2^). The effect of ED was significant (*P* < 0.05) on the relative abundance of 3 bacterial genera representing more than 1% of the microbiota (*Streptococcus*, *Coprococcus* and a *Lachnospiraceae* genus); but these effects were not significant when considering *P*-values corrected for false discovery rate (Additional file 1). Overall, ED did not induce major modifications of the fecal microbiota at the end of the post-weaning period.

## Discussion

The health status and growth performance of pigs were analyzed to evaluate the effects of a dietary supplementation with a blend of functional AA (Gln, Arg, Cys2 and BCAA) and grape extract polyphenols during the post-weaning period on pig capacity to cope with weaning and a subsequent inflammatory challenge caused by poor hygiene of housing conditions. Modulatory effects on gut microbiota composition and metabolic activity, and on epithelial homeostasis in intestinal organoids were recently reported in weaned pigs fed AA and polyphenols-supplemented diet [11]. Therefore, we hypothesized that pigs fed AAP diet during the post-weaning period would be healthier and more robust at the end of post-weaning to cope with an inflammatory challenge applied in the beginning of the growing period, when they were not fed anymore this supplemented diet. Our major and original findings is that AAP diet delivered during the post-weaning period supported weaning transition and prevented the detrimental effects of poor hygiene conditions in the beginning of the growing period.

During the post-weaning period, pigs fed AAP diet had lower plasma haptoglobin concentrations, a trend to lower values of dROM and ISO, and greater concentrations of vitamins E and A compared to CNT pigs. Haptoglobin is a major acute phase protein in pigs and an increase in its plasma concentration is an indicator of inflammation and/or infection [13]. Weaning is known to increase the concentration of haptoglobin in the plasma [2, 12]. In pigs, greater plasma concentrations of haptoglobin after than before weaning were considered as inflammatory [2, 14] and/or stress [14] responses related to weaning. Furthermore, dROM and ISO are indicators of oxidative stress [15]. Higher dROM and haptoglobin concentrations, and lower concentrations of vitamins E and A observed in pigs after weaning are explained by inflammation of the intestinal mucosa and increase oxidative stress as consequence of weaning-induced stress [15–17]. Vitamins E and A are antioxidant molecules [15]. Vitamin E is mainly related to hydroperoxyl radical scavenging [18] and vitamin A acts via hydrogen atom donating [19]. Lower plasma concentrations of vitamins E and A were observed in pigs 5 days after weaning wherein vitamin E remained low until 19 days after weaning [15]. These findings were associated with a greater production of free radicals, which in turn are neutralized by antioxidant molecules (such as vitamins E and A) leading to a decrease in their concentrations [15]. Therefore, the lower haptoglobin and the trends for greater vitamins E and A concentrations in pigs fed AAP during post-weaning may be indicative of a lower inflammatory status associated with greater antioxidant potential. Furthermore, it can be hypothesized that greater plasma concentrations of both vitamins in AAP pigs may be a result of their lower utilization, probably because of the supply of polyphenols acting as exogenous antioxidants. Weaning also impairs the intestinal barrier that can result in post-weaning diarrhea [20]. More pigs were observed with than without diarrhea in CNT pigs while no difference was observed for AAP pigs, suggesting a protective effect of AAP. Diamine oxidase is an indicator of  digestive integrity [21]. In humans, an increase in plasma DAO concentrations reflects impairment of intestinal morphology and lower nutrient absorption [21]. Therefore, the lower concentrations of DAO in AAP compared to CNT pigs suggest that AAP pigs had better mucosa integrity and effective barrier function, which may indicate that AAP dietary supplementation plays a protective role in gut health during the post-weaning period. Accordingly, Beaumont et al. [11] reported that a mix of AA and polyphenols upregulated the gene expression of indicators of epithelial differentiation and reduced the expression of inflammatory indicators in pig intestinal organoids. In the same study, supplementation of weaned piglets with AA and polyphenols induced modulation of the gut microbiota by increasing the abundance of *Lactobacillaceae* in the jejunum while decreasing the abundance of the *Proteobacteria* in the caecum [11]. In contrast, in the current study, AAP supplementation had no major effect on the fecal microbiota 6 weeks after weaning. Difference between the two studies might be explained by the gut segment studied (jejunum/caecum versus fecal samples). Indeed, supplemented AA and polyphenols are probably found in greater concentrations in the middle intestine where they exert their effect on microbiota. Moreover, we analyzed the microbiota 6 weeks after weaning while the previous study analyzed samples collected 2 weeks after weaning, when the microbiota is still adapting to the new diet and environment. Since pigs from both diets started the experiment with similar BW and health indicators, the better health status observed for AAP pigs during the post-weaning period could be explained by AA and grape polyphenols biological functions. Among the AA in the blend, Cys2 and Arg are strongly associated with increase in antioxidant capacity [22] and/or regulating the inflammation in weaned pigs [23, 24]. Glutamine is a gut-trophic nutrient, whose effects are improvement of intestinal morphology and permeability, lower oxidative damage, and higher enterocyte proliferation in pigs [25, 26]. Dietary supplementation with 1% L-Gln enhanced intestinal oxidative-defense capacity of weaned pigs, demonstrated by greater jejunal concentrations of glutathione (GSH; 29%) and reduced jejunal concentrations of oxidized glutathione (GSSG; -18%) compared to non-supplemented pigs [26]. Besides AA, grape polyphenols are also related with antioxidant and anti-inflammatory effects [27]. Accordingly, pigs fed polyphenols-supplemented diet during the post-weaning period had greater antioxidant capacity evidenced by expression of enzymatic defense system [10]. To summarize, these nutrients and nutrient-related metabolites promote health in response to stressful stimuli such as weaning [5, 11].

In the current study, we used a model of poor hygiene of housing conditions to induce a moderate inflammatory state. This model is known to reduce pig growth performance and to increase plasma haptoglobin concentration and granulocyte count in the blood [28]. Furthermore, lower growth rate and greater prevalence of diarrhea were observed in pigs housed in poor compared to good hygiene conditions [14, 29]. In addition to greater WBC and blood granulocyte count, FCR and the prevalence of diarrhea were higher in pigs housed in poor conditions compared to pigs in good conditions. Still 11 weeks after the end of the challenge, pigs previously housed in poor conditions tended to be lighter (4 kg/animal) than those previously housed in good hygiene, indicating that pigs leaving poor hygiene conditions did not completely recover from the challenge as already shown by Chatelet et al. [30]. Interestingly, during the challenge period, most of the effects of poor hygiene were reported in CNT pigs only. Growth rate, haptoglobin and vitamin E concentrations in pigs previously fed AAP diet during the post-weaning period were not affected by poor hygiene conditions. As above-mentioned, AAP pigs had lower inflammatory responses and greater antioxidant capacity in the post-weaning period. Recently, it has been shown that diet supplementation with the AA L-threonine, DL-methionine, and L-tryptophan improved antioxidant defense system in post-weaned pigs and prepared them better to cope with a subsequent *Salmonella* challenge [31]. These authors observed that an adaptation period with AA-supplemented diet reduced the inflammatory responses (increase albumin and decrease haptoglobin plasma concentrations), which was accompanied by reducing colonization of the inoculated *Salmonella* [31]. Furthermore, Arg supplementation in a milk replacer diet during the pre-weaning period supported pigs’ weaning transition and improved intestinal morphology (greater villus height, villous area, and relative intestine weight) in post-weaned pigs [32]. Accordingly, our results showed that the positive effects of AA and grape polyphenols supplementation extend beyond the supplemented period and may help to support health and growth performance later on.

## Conclusions

Our results show an improvement of the capacity of AAP pig to cope with the weaning transition as evidenced by lower concentrations of the digestive integrity marker, greater levels of antioxidant markers at the end of the weaning period, and lower prevalence of diarrhea. The absence of negative effects of hygiene challenge in AAP pigs indicated that the beneficial effects with 0.2% AAP dietary supplementation persisted after the post-weaning period and resulted in a greater ability of pigs to cope with poor hygiene conditions. These findings support the development of nutritional strategies potentially applicable in the field to improve health of pigs during critical periods.

## Methods

### Experimental design

The study was conducted in accordance with ARRIVE guidelines (https://arriveguidelines.org). The experiment was carried out at INRAE (UE3P, Saint-Gilles, France doi.org/10.15454/1.5573932732039927E12), in compliance with the Directive 2010/63/UE on animal experimentation. The experimental protocol was approved by the regional Ethical Committee in Animal Experiment of Rennes (France) and by the French Ministry of Higher Education, Research and Innovation (authorization APAFIS#23735–2020012214413172). Eighty male and female Piétrain x Large-White x Landrace pigs born from 30 sows belonging to INRAE UE3P were selected and weaned at 28 day (8.79 kg BW; SEM: 0.12 kg). The experiment was conducted in two batches of 40 animals born at six weeks of interval. The same experimental design was replicated in both batches. For each batches, the experiment lasted 20 weeks and was divided into 3 periods: post-weaning (from week 0 to 6); challenge (from week 7 to 9); and finishing (from week 10 to 20).

During the post-weaning period, pigs were fed a control standard diet supplemented or not with 0.2% of a blend of functional AA and grape polyphenols (AAP and CNT diets, respectively). The blend contained L-arginine, L-cystine, L-leucine, L-valine, L-isoleucine and L-glutamine (in a weight ratio 35:15:8:8:8:20) and 40 ppm of polyphenol-rich grape extract. The amino acids composing the blend were the same as the study by Beaumont et al. [11] plus glutamine known to exert trophic effect on the intestinal tract of weaned pigs [25]. The dose of grape extract polyphenols was close to the lowest dose that reduced the prevalence of diarrhea in weaned pigs in the study of Hao et al. [10]. The grape extract was obtained from seeds and skins (85 and 15%, respectively) and contains around 70% of polyphenols on dry material. Proanthocyanidins, class of polyphenols from the flavonoid family, are the predominant polyphenols in grape extract. The composition and calculated nutrient content of diets provided during the pre-starter (from week 0 to 1; post-weaning) and starter (from week 2 to 6; post-weaning) phases are presented in the Additional file 2.

At the end of W6, pigs were transferred to a growing unit where 50% of pigs fed AAP and CNT diets were housed in good and the other 50% in poor hygiene conditions for three-weeks (challenge period). At the end of challenge period (at W9), regardless of experimental diet and hygiene of housing conditions, pigs were transferred in another room within the same experimental growing unit (finishing period). Pigs were slaughtered 20 weeks after weaning.

### Animals housing and feeding

During the post-weaning period, pigs were group housed in slatted floor pens (145 × 270 cm, *n* = 5 pigs/pen) equipped with a feeder and a nipple water drinker. During the challenge period, pigs were housed in individual pens (85 × 265 cm) equipped with a feed dispenser and a nipple drinker. All pigs were fed the same standard diet formulated to meet the nutritional requirements of growing pigs (9.47 MJ of net energy/kg, starch 44.2%; fat 3.1%; crude protein 15.3% and 8.3 g of standardized ileal digestible lysine (SID Lys), SID Lys/kg). The model of good and poor hygiene condition study was described in detail in previous studies [13, 28]. To summarize, poor housing conditions consisted of no cleaning nor sanitation of the room after a previous occupation by non-experimental pigs, whereas for good hygiene conditions, the room was cleaned daily, in addition to the application of optimal aeration rate and temperature and strict biosecurity precautions. The staff put clean boots and changed clothes every time before entering the room.

During the finishing period, consecutive pens (330 × 545 cm, *n* = 15 pigs/pen) hosted alternatively pigs previously housed in good and poor hygiene conditions. Pigs were fed with the same standard finishing diet (9.69 MJ of net energy/kg, starch 45.8%; fat 3.0%; crude protein 16.5% and 9.3 g of SID Lys/kg). Throughout the entire experimental period, the photoperiod was fixed to 12 h of artificial light (0600 to 1800 h) in temperature-controlled rooms. The ambient temperature was recorded every 30 min using 2 data loggers (LoRa® SPY, JRI, Chicago, US) located in the room’s extremities and at half the height of the body of the animals. Feed and water were provided ad libitum.

### Growth performance and health evaluation

Throughout the trial, each animal was individually inspected and diarrhea, coughing, lameness, and any other clinical signs were recorded two (post-weaning period) or three (challenge period) times per week. To assess the prevalence of diarrhea, a scale of three levels was used (0 = normal, 1 = soft feces and 2 = diarrhea). During the challenge period, measurements of air concentration in ammonia, carbon dioxide, and hydrogen sulfide in both hygiene conditions were carried out at 30 cm above floor with a manual pump (Drager, Draeger Accuro Manual Tube Pump) at week 7, week 8 and week 9. These measurements were done between 0800 and 0900 h in two locations of the rooms.

After an overnight fasting, pigs were weighed at week 0 (just before the pigs weaning), one and six weeks after the weaning, and at the end of challenge and finishing periods. Feed consumption was estimated weekly during the post-weaning and challenge periods as the difference between allocated feed and refusals.

### Blood and fecal sampling

The days of BW recording, blood samples were collected between 0800 and 1000 h via jugular punctures into heparinized and ethylenediaminetetraacetic acid (EDTA) tubes. Pigs were manually restrained on dorsal decubitus at W0, W1 and W6 and using a snout rope at W9. The time of snout restraining was carefully checked not to exceed 2 min to limit pain. When no blood was collected within these 2 min, the sample was considered as missing. Samples were kept on ice, except for blood cell count assessment, and were centrifuged (3000 rpm, 4 °C, 15 min). Plasma was aliquoted after collecting and then kept at -80˚C or -20˚C before analyses. Fecal samples were collected one day before the blood sampling performed at W6 to analyze the microbiota composition. Fecal samples were immediately frozen in liquid nitrogen after collecting and then kept at -80ºC until analysis.

### Blood analyses

White blood cell count and plasma DAO concentrations were determined from blood collected on EDTA. The other plasma variables were measured from blood collected on heparin. Total number of WBC and the differential count of granulocytes (eosinophils, basophils, and neutrophils) were measured with a hematology automated cell counter calibrated for pigs (MS9®; Melet Schloesing Laboratories, Osny, France). Automated enzymatic methods using a multianalyzer apparatus (Konelab20i, Thermo Electron Corporation, Cergy, France) were used to determine plasma concentrations of dROM and biological antioxidant potential (BAP) (dROM and BAP tests, references MC003 and MC437, respectively; Diacron, Grosseto, Italy). Oxidative stress index was calculated as the dROM to BAP ratio as previously described [13]. Plasma concentrations of vitamins E and A were assayed by liquid chromatography (HPLC, Waters Alliance) on a dedicated column (Chromsystems, Germany). Quantitative sandwich ELISA tests (Catalog Number MBS9718395) were used to measure DAO concentrations.

#### Microbiota analysis

Fecal microbiota was analyzed by 16S rRNA gene sequencing as described before [10]. Briefly, DNA was extracted from fecal samples using Quick-DNA Fecal/Soil Microbe 96 Kit (ZymoResearch, Irvine, CA) and PCR amplicons of the V3-V4 region were sequenced by MiSeq Illumina Sequencing. Reads were deposited in the National Center for Biotechnology Information Sequence Read Archive (SRA accession: PRJNA881232). Sequences were analyzed with the ‘FROGS’ bioinformatic pipeline, following the standard guidelines [33]. Amplicons were selected based on their size (350 – 500 nucleotides). Clustering was performed with Swarm using an aggregation distance of 1. Chimera was removed and operational taxonomic units (OTUs) with an abundance lower than 0.005% of the total of sequences were excluded. The taxonomic affiliation of OTUs was performed with the database 16S SILVA 138.1 with a pintail of 100. The mean number of reads was 15 902 and rarefaction was performed for alpha and beta diversity analysis to 7 975 reads per sample.

#### Calculations and statistical analysis

The ADG (g/day) and ADFI (g/day) were calculated from the BW gain and the feed consumption, respectively. Feed conversion ratio was calculated as the feed-to-gain ratio (kilogram of feed consumed per kilogram BW).

Statistical analyses were carried out separately for each period using the SAS software (version 9.3, SAS Inst. Inc., Cary, NC). The presence of outliers was evaluated through the residual analysis of data and by daily records of anomalies. Through UNIVARIATE procedure, BoxCox and Shapiro–Wilk tests were used to check homogeneity of the variances and normality of the studentized residuals, respectively. The data were analyzed using linear mixed effects models (MIXED procedure) and were presented as least squares means and pooled residual standard errors. For the post-weaning period, the model included the fixed effects of ED, sex, repetition, and their interactions. For the challenge and finishing periods, the Hyg effect and interactions with ED were included as main effects. The animal was considered as a random error. The individual pig was considered as experimental unit, except for ADFI during the post-weaning where the pen was used as experimental unit. When there was an interaction between ED and Hyg (*P* ≤ 0.05), adjusted means were compared using a post hoc Bonferroni test. The prevalence of diarrhea was compared between the experimental diets (post-weaning period) and experimental diets and hygiene conditions (challenge period) using a χ^2^ test (PROC FREQ, SAS). A difference was considered as significant when *P* ≤ 0.05, whereas 0.05 < *P* ≤ 0.10 was discussed as a trend.

The microbiota analysis was performed using the R software (version 4.2.0) with the phyloseq package (version 1.40.0). Bray–Curtis distances were calculated on the rarefied count matrix and analyzed with PERMANOVA. Microbiota richness, Shannon, and Inverse Simpson α-diveristy indexes were calculated on the rarefied count matrix. When the relative abundance was lower than 0.05%, the OTUs were removed from the statistical analysis. Microbiota richness, Shannon and Inverse Simpson α-diveristy indexes and bacterial abundances at the phylum, family and genus levels were analyzed by ANOVA with linear mixed models including the fixed effects of experimental diet, sex and their interactions. Random effects included the litter, the pen and the repetition. Bacterial relative abundances were square root transformed before analysis. False discovery rate adjustments were used for multiple testing (family and genus levels).

## Supplementary Information

**Additional file 1.**

**Additional file 2.**

## Data Availability

For microbiota analyses, reads were deposited in the National Center for Biotechnology Information Sequence Read Archive (SRA accession: PRJNA881232). Other individual data used/analyzed in this study are available from the corresponding author on reasonable request.

## Abbreviations

W: Week
AA: Amino acids
AAP: Blend of functional amino acids and grape polyphenols
BCAA: Branched-chain amino acids
Lys: Lysine
Arg: Arginine
Cys2: Cystine, Gln: Glutamine
CNT: Control
BW: Body weight
ADFI: Average daily feed intake
ADG: Average daily gain
FCR: Feed Conversion Rate
dROM: Diacron-reactive Oxygen Metabolites
BAP: Biological Antioxidant Potential
OSI: Oxidative Stress Index
OTUs: Operational Taxonomic Units
CARRU: Carratelli Unit
HPLC: High Pressure Liquid Chromatography
EDTA: Ethylenediaminetetraacetic Acid
DAO: Diamine Oxidase
WBC: White Blood Cells
ED: Experimental diet
Hyg: Hygiene condition
ED × Hyg: Interaction between ED and Hyg
SEM: Standard error of the mean
P or *P*-value: Probability value
