# Supplementary material for: A blend of functional amino acids and grape polyphenols improves the pig capacity to cope with an inflammatory challenge caused by poor hygiene of housing conditions
Source: BMC Vet Res. 2023 Jan 30;19:25. doi: 10.1186/s12917-023-03580-w (PMC9887908; doi:10.1186/s12917-023-03580-w)
Supplement: Supplementary file 1 — Additional file 1. [file 12917_2023_3580_MOESM1_ESM.docx]

**Additional file 1**

Table S1. Fecal microbiota of CNT and AAP pigs at week 6.

|  | Mean | |  | SEM^1^ | | P-value^2^ | P-adjusted^3^ |
| --- | --- | --- | --- | --- | --- | --- | --- |
|  | CNT | AAP |  | CNT | AAP |  |  |
| **Diversity** |  |  |  |  |  |  |  |
| Richness | 439.08 | 434.92 |  | 6.79 | 7.69 | 0.63 |  |
| Shannon | 4.51 | 4.46 |  | 0.03 | 0.04 | 0.26 |  |
| InvSimpson | 35.46 | 32.95 |  | 1.50 | 1.85 | 0.34 |  |
| **Phylum** (relative abundance)^4^ |  |  |  |  |  |  |  |
| Firmicutes | 65.24 | 67.53 |  | 1.08 | 1.21 | 0.16 |  |
| Bacteroidota | 33.59 | 31.14 |  | 1.10 | 1.18 | 0.12 |  |
| **Family** (relative abundance)^5^ |  |  |  |  |  |  |  |
| Prevotellaceae | 32.30 | 29.64 |  | 1.14 | 1.23 | 0.12 | 0.58 |
| Lachnospiraceae | 22.17 | 20.82 |  | 0.75 | 0.72 | 0.16 | 0.58 |
| Lactobacillaceae | 20.32 | 23.68 |  | 1.47 | 1.82 | 0.15 | 0.58 |
| Ruminococcaceae | 8.28 | 7.51 |  | 0.31 | 0.41 | 0.18 | 0.58 |
| Clostridiaceae | 3.39 | 3.78 |  | 0.32 | 0.72 | 0.63 | 0.87 |
| Butyricicoccaceae | 2.86 | 2.71 |  | 0.14 | 0.14 | 0.33 | 0.74 |
| Veillonellaceae | 1.84 | 1.61 |  | 0.12 | 0.11 | 0.22 | 0.58 |
| Streptococcaceae | 1.81 | 2.65 |  | 0.33 | 0.32 | **0.02** | 0.38 |
| Peptostreptococcaceae | 1.58 | 1.55 |  | 0.13 | 0.21 | 0.94 | 0.94 |
| Oscillospiraceae | 1.47 | 1.57 |  | 0.11 | 0.13 | 0.56 | 0.87 |
| **Genus** (relative abundance)^6^ |  |  |  |  |  |  |  |
| Prevotella_9 | 23.41 | 20.83 |  | 0.98 | 1.10 | 0.06 | 0.39 |
| Lactobacillus | 15.27 | 17.00 |  | 1.09 | 1.19 | 0.26 | 0.74 |
| Limosilactobacillus | 4.97 | 6.57 |  | 0.42 | 0.66 | 0.07 | 0.42 |
| Blautia | 4.34 | 3.93 |  | 0.26 | 0.17 | 0.25 | 0.74 |
| Faecalibacterium | 4.05 | 3.39 |  | 0.24 | 0.25 | 0.05 | 0.39 |
| Roseburia | 3.58 | 3.46 |  | 0.22 | 0.22 | 0.63 | 0.95 |
| Prevotella | 3.12 | 2.93 |  | 0.19 | 0.22 | 0.46 | 0.94 |
| UCG-008 | 2.59 | 2.49 |  | 0.14 | 0.14 | 0.48 | 0.95 |
| Clostridium sensu stricto 1 | 2.43 | 2.80 |  | 0.31 | 0.65 | 0.98 | 0.99 |
| Ruminococcus | 1.99 | 1.74 |  | 0.11 | 0.11 | 0.20 | 0.71 |
| Prevotella_7 | 1.98 | 2.20 |  | 0.35 | 0.40 | 0.80 | 0.95 |
| Subdoligranulum | 1.97 | 2.11 |  | 0.07 | 0.16 | 0.70 | 0.95 |
| Dialister | 1.84 | 1.61 |  | 0.12 | 0.11 | 0.22 | 0.71 |
| Streptococcus | 1.81 | 2.65 |  | 0.33 | 0.32 | **0.02** | 0.39 |
| Lachnospiraceae_multiaffiliation | 1.78 | 1.43 |  | 0.13 | 0.14 | **0.01** | 0.39 |
| Coprococcus | 1.22 | 1.39 |  | 0.06 | 0.06 | **0.04** | 0.39 |
| Terrisporobacter | 1.19 | 1.21 |  | 0.12 | 0.20 | 0.83 | 0.95 |
| Prevotellaceae NK3B31 group | 1.02 | 0.96 |  | 0.11 | 0.11 | 0.87 | 0.95 |

^1^ Standard error of the mean.

^2^ Probability value for the effect of experimental diets (P ≤ 0 .05 are indicated in bold).

^3^ Probability value for the effect of experimental diets adjusted for false discovery rate.

^4,5,6^ For each taxonomic rank (phylum, family, and genus), bacterial groups with a relative abundance > 1% are presented.
