# Supplementary material for: A blend of functional amino acids and grape polyphenols improves the pig capacity to cope with an inflammatory challenge caused by poor hygiene of housing conditions
Source: BMC Vet Res. 2023 Jan 30;19:25. doi: 10.1186/s12917-023-03580-w (PMC9887908; doi:10.1186/s12917-023-03580-w)
Supplement: Supplementary file 2 — Additional file 2. [file 12917_2023_3580_MOESM2_ESM.docx]

**Additional file 2**

Table S2. Ingredients and calculated composition of the experimental diets.^1^

|  | Pre-starter | | |  | Starter | |
| --- | --- | --- | --- | --- | --- | --- |
| Experimental diets | CNT | | AAP |  | CNT | AAP |
| Ingredient composition as-fed basis, % | |  |  |  |  |  |
| Wheat | | 33.15 | 33.08 |  | 24.40 | 24.35 |
| Barley | | 25.19 | 25.14 |  | 25.13 | 25.08 |
| Soybean, Grain | | 8.06 | 8.04 |  | 0.00 | 0.00 |
| Soybean, Meal | | 0.00 | 0.00 |  | 19.97 | 19.93 |
| Soybean, Cake | | 11.06 | 11.04 |  | 0.00 | 0.00 |
| Soy protein concentrate | | 2.52 | 2.51 |  | 0.00 | 0.00 |
| Corn | | 0.00 | 0.00 |  | 20.10 | 20.06 |
| Rapeseed | | 0.00 | 0.00 |  | 3.02 | 3.01 |
| Beet pulp | | 0.00 | 0.00 |  | 2.51 | 2.50 |
| Vegetable oil | | 0.77 | 0.77 |  | 1.00 | 1.00 |
| Lactose | | 13.60 | 13.57 |  | 0.00 | 0.00 |
| Calcium carbonate | | 1.04 | 1.04 |  | 1.21 | 1.21 |
| Monocalcium phosphate | | 0.69 | 0.69 |  | 0.61 | 0.61 |
| Salt | | 0.30 | 0.30 |  | 0.40 | 0.40 |
| Potato protein concentrate | | 1.00 | 1.00 |  | 0.00 | 0.00 |
| L-Lysine^2^ | | 0.57 | 0.57 |  | 0.62 | 0.62 |
| Methionine^3^ | | 0.24 | 0.24 |  | 0.10 | 0.10 |
| L-Threonine | | 0.25 | 0.25 |  | 0.15 | 0.15 |
| L-Tryptophan | | 0.09 | 0.09 |  | 0.13 | 0.13 |
| L-Valine | | 0.16 | 0.16 |  | 0.04 | 0.04 |
| Vitamin and mineral premix^4^ | | 0.50 | 0.50 |  | 0.50 | 0.50 |
| Phytase and organic acids | | 0.81 | 0.81 |  | 0.11 | 0.11 |
| Blend of functional AA and grape polyphenols | | 0.00 | 0.20 |  | 0.00 | 0.20 |
| Calculated composition^5^ | |  |  |  |  |  |
| Net energy, MJ/kg | | 10.57 | 10.57 |  | 9.55 | 9.55 |
| Crude protein, % | | 18.01 | 18.06 |  | 18.40 | 18.70 |
| Free lysine, % | | 0.47 | 0.47 |  | 0.33 | 0.33 |

^1^ Pigs received a CNT or AAP diet during pre-starter (from week 0 to 1) and starter (from week 2 to 6) phases. The AAP diet consisted of a standard diet (CNT) supplemented with 0.2% a blend of functional amino acids (L-arginine, L-cystine, L-leucine, L-valine, L-isoleucine, and L-glutamine) and grape polyphenols.

^2^ L-lysine HCL (pre-starter) and liquid lysine; 50.0% of L-lysine (starter).

^3^ L-methionine (pre-starter) and DL-methionine (starter).

^4^ Mineral vitamin supplement (per kg of diet): Vit. A (2.000.000 UI); Vit. D3 (400.000 UI); Vit. E (4.000 mg); Vit. K3 (870 mg); Vit. B1 (400 mg); Vit. B2 (1.000 mg); Vit. B6 (1.000 mg); Vita. C (8.000 mg); Niacin (4.000 mg); Pantothenic acid (2.000 mg); Folic acid (200 mg); Biotin (40 mg); Vit. B12 (6.0 mg); Copper (4.000 mg); Iodine (120 mg); Manganese (8.000 mg); Selenium (60 mg); Zinc (20.000 mg); Ferrous sulphate (20.000 mg); Choline chloride (160.000 mg); Butylated Hydroxyanisole (75 mg); and Butylated Hydroxytoluene (75 mg).

^5^ The calculated values (%) for L-arginine, L-cystine, L-leucine, L-isoleucine, and L-glutamine for CNT diet in both phases were, respectively: 0.04; 0.01; 0.003; 0.004; and 0.05. For AAP diet, the values were, respectively: 0.10; 0.04; 0.02; 0.02; and 0.05. For L-valine the values were in pre-starter phase: 0.18 (CNT diet) and 0.20 (AAP diet); and in starter phase 0.03 (CNT diet) and 0.04 (AAP diet).
